# Tetra­kis(μ-4-methyl­benzoato-κ*O*:*O*′)bis­{[4-(dimethyl­amino)pyridine-κ*N* ^1^]zinc(II)}

**DOI:** 10.1107/S1600536809027603

**Published:** 2009-07-18

**Authors:** Lin-Shan Bai, Xin-Hua Liu, Zhu-Ping Xiao

**Affiliations:** a Key Laboratory of Anhui Educational Department, Anhui University of Technology, Maanshan 243002, People’s Republic of China; b State Key Laboratory of Pharmaceutical Biotechnology, Nanjing University, Nanjing 210093, People’s Republic of China

## Abstract

In the centrosymmetric title binuclear complex, [Zn_2_(C_8_H_7_O_2_)_4_(C_7_H_10_N_2_)_2_], the Zn atoms [Zn⋯Zn = 3.0287 (6) Å] are bridged by four 4-methyl­benzoate ligands. The four nearest O atoms around each Zn^II^ atom form a distorted square-planar arrangement with the distorted square-pyramidal coordination completed by the pyridine N atom of the 4-(dimethyl­amino)pyridine ligand. In the crystal structure, weak inter­molecular C—H⋯O inter­actions link the mol­ecules into infinite chains. The chains are further linked by weak C—H⋯π inter­actions, forming a three-dimensional network.

## Related literature

For potential applications of organometallic complexes, see: Sommerfeldt *et al.* (2008 ▶); Huang *et al.* (2007 ▶); Neville *et al.* (2008 ▶). Zinc derivatives are used in photodynamic therapy because of their unique photosensitizing properties, see: Tabata *et al.* (2000 ▶); Shi *et al.* (2008 ▶); Xiao *et al.* (2008 ▶); Yang *et al.* (2008 ▶). For comparative bond lengths, see: Halcrow *et al.* (2000 ▶); For related structures, see: Yang *et al.* (2004 ▶); You *et al.* (2003 ▶, 2004 ▶); Wang *et al.* (2009 ▶).
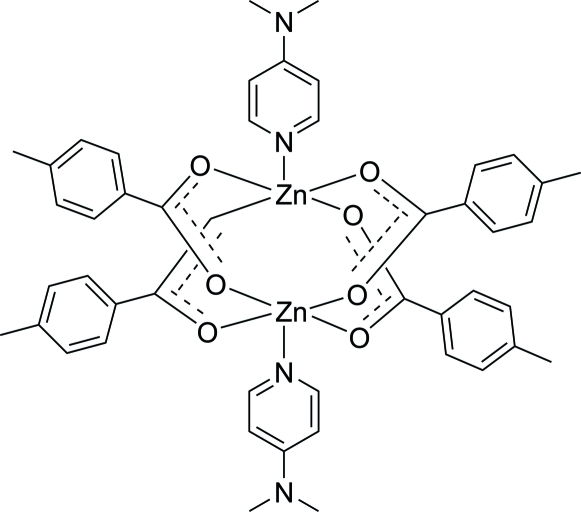


## Experimental

### 

#### Crystal data

[Zn_2_(C_8_H_7_O_2_)_4_(C_7_H_10_N_2_)_2_]
*M* *_r_* = 915.66
Monoclinic, 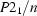

*a* = 8.9311 (18) Å
*b* = 9.967 (2) Å
*c* = 24.756 (5) Å
β = 90.64 (3)°
*V* = 2203.5 (8) Å^3^
*Z* = 2
Mo *K*α radiation
μ = 1.15 mm^−1^
*T* = 294 K
0.30 × 0.20 × 0.20 mm

#### Data collection

Bruker APEXII CCD area-detector diffractometer
Absorption correction: ψ scan (*SADABS*; Sheldrick, 1996 ▶) *T*_min_ = 0.758, *T*_max_ = 0.792
12095 measured reflections
4326 independent reflections
3432 reflections with *I* > 2σ(*I*)
*R*_int_ = 0.020

#### Refinement

*R*[*F* ^2^ > 2σ(*F* ^2^)] = 0.031
*wR*(*F* ^2^) = 0.085
*S* = 1.03
4326 reflections
275 parameters
H-atom parameters constrained
Δρ_max_ = 0.21 e Å^−3^
Δρ_min_ = −0.28 e Å^−3^

### 

Data collection: *SMART* (Bruker, 2000 ▶); cell refinement: *SAINT* (Bruker, 2000 ▶); data reduction: *SAINT*; program(s) used to solve structure: *SHELXS97* (Sheldrick, 2008 ▶); program(s) used to refine structure: *SHELXL97* (Sheldrick, 2008 ▶); molecular graphics: *SHELXTL* (Sheldrick, 2008 ▶); software used to prepare material for publication: *SHELXTL*.

## Supplementary Material

Crystal structure: contains datablocks global, I. DOI: 10.1107/S1600536809027603/hk2733sup1.cif

Structure factors: contains datablocks I. DOI: 10.1107/S1600536809027603/hk2733Isup2.hkl

Additional supplementary materials:  crystallographic information; 3D view; checkCIF report

## Figures and Tables

**Table:** 

O1—Zn1^i^	2.0564 (16)
O2—Zn1	2.0626 (15)
O3—Zn1	2.0438 (15)
O4—Zn1^i^	2.0320 (16)
N1—Zn1	2.0160 (16)

**Table:** 

O3—Zn1—O1^i^	86.16 (7)
O3—Zn1—O2	88.75 (6)
O4^i^—Zn1—O1^i^	89.51 (7)
O4^i^—Zn1—O2	86.67 (7)
O4^i^—Zn1—O3	157.18 (6)
N1—Zn1—O1^i^	99.01 (7)
N1—Zn1—O2	103.64 (7)
N1—Zn1—O3	100.83 (7)
N1—Zn1—O4^i^	101.98 (7)

Symmetry code: (i) 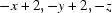.

**Table 2:** Hydrogen-bond geometry (Å, °)

*D*—H⋯*A*	*D*—H	H⋯*A*	*D*⋯*A*	*D*—H⋯*A*
C21—H21*C*⋯O3^ii^	0.96	2.54	3.484 (3)	168
C23—H23*B*⋯*Cg*1^iii^	0.96	2.99	3.925 (4)	165

Symmetry codes: (ii) 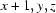; (iii) 
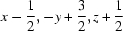
. *Cg*1 is the centroid of atoms C3–C8.

**Fig. 1.**
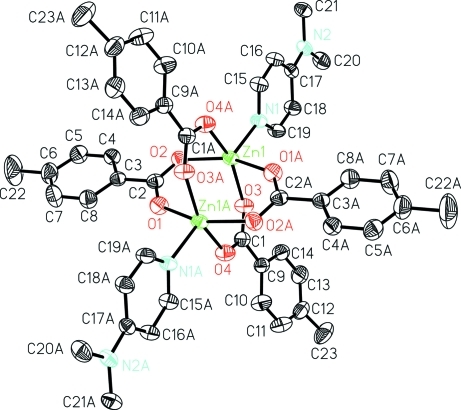
The molecular structure of the title molecule with the atom-numbering scheme. Displacement ellipsoids are drawn at the 30% probability level [symmetry code: (A) 2 - x, 2 - y, -z]. H atoms have been omitted for clarity.

**Fig. 2.**
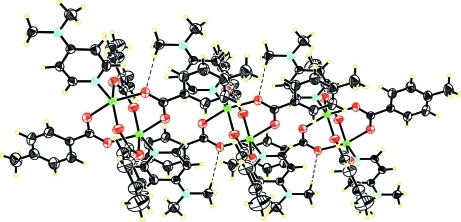
The chain formed through C—H···O intermolecular hydrogen bonds. Hydrogen bonds are shown as dashed lines.

**Fig. 3.**
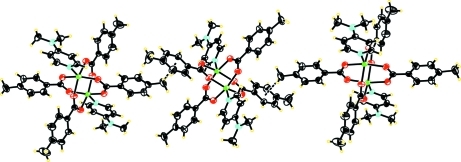
A partial packing diagram, with C—H···π contacts shown as dashed lines.

## supplementary crystallographic information

### Comment

Numerous organometallic complexes have been designed for a number of potential applications, such as in synthetic chemistry (Sommerfeldt *et al.*, 2008), as luminescence materials (Huang *et al.*, 2007) and as magnetic materials (Neville *et al.*, 2008). Zinc derivatives are particularly interesting owing to their unique photosensitizing properties for photodynamic therapy (Tabata *et al.*, 2000; Shi *et al.*, 2008; Xiao *et al.*, 2008; Yang *et al.*, 2008;), magnetic circularly polarized luminescence (MCPL) and magnetic circular dichroism (MCD) spectra. We have reported the structures of a few zinc(II) complexes (Yang *et al.*, 2004; You *et al.*, 2003, 2004). As an extension of our work on the structural characterizations of zinc compounds, we report herein the crystal structure of the title compound.

The title compound is a binuclear compound (Fig. 1), consisting of four 4-methylbenzoato and two 4-(*N*,*N*-diamino)pyridine ligands. It has a centre of symmetry. The 4-(*N*,*N*-diamino)pyridine ligands are coordinated to Zn atoms through pyridine N atoms only. The 4-methylbenzoato groups act as bridging ligands. The Zn···ZnA distance is 3.0287 (6) Å and the N1-Zn1···Zn1A angle is 169.82 (5) [symmetry code: (A) 2 - x, 2 - y, -z]. The four O atoms of the bridging 4-methylbenzoato ligands around each Zn atom form a distorted square plane (Table 1). A distorted square-pyramidal arrangement around each Zn atom is completed by the pyridine N atom of 4-(*N*,*N*-diamino)pyridine ligand (Table 1). The dihedral angle between plane through Zn1, O1, O2, C2, Zn1A, O1A, O2A, C2A and the plane through Zn1, O3, O4, C1, Zn1A, O3A, O4A, C1A is 87.931 (24) °. The Zn-O bonds are in the range of 2.0320 (16)-2.0626 (15) Å, and are in accordance with the corresponding values in a similar compound (Wang *et al.*, 2009). The Zn1-N1 [2.0160 (16) Å] bond is significantly shorter than the corresponding reported values (Halcrow *et al.*, 2000).

In the crystal structure, weak intermolecular C-H···O interactions (Table 2) link the molecules into infinite chains (Fig. 2), in which they are further linked by weak C—H···π interactions (Table 2) to form a three-dimensional network (Fig. 3).

### Experimental

For the preparation of the title compound, zinc oxide (0.5 mmol) and 4-methylbenzoic acid (1 mmol) were dissolved in aqueous ammonia (10 ml, 30%,) and then, 4-(*N*,*N*-dimethylamino)pyridine (0.5 mmol) was added. The resulting solution was stirred at room temperature and then filtered. Crystals suitable for X-ray analysis were obtained after 13 to 15 d by volatilization of the solvents.

### Refinement

H atoms were positioned geometrically with C-H = 0.93 and 0.96 Å, for aromatic and methyl H atoms, respectively, and constrained to ride on their parent atoms, with U_iso_(H) = xU_eq_(C), where x = 1.5 for methyl H and x = 1.2 for aromatic H atoms.

### Crystal data

**Table:**

[Zn_2_(C_8_H_7_O_2_)_4_(C_7_H_10_N_2_)_2_]	*F*(000) = 952
*M**_r_* = 915.66	*D*_x_ = 1.380 Mg m^−^^3^
Monoclinic, *P*2_1_/*n*	Mo *K*α radiation, λ = 0.71073 Å
Hall symbol: -P 2yn	Cell parameters from 3875 reflections
*a* = 8.9311 (18) Å	θ = 1.8–29.5°
*b* = 9.967 (2) Å	µ = 1.15 mm^−^^1^
*c* = 24.756 (5) Å	*T* = 294 K
β = 90.64 (3)°	Block, colorless
*V* = 2203.5 (8) Å^3^	0.30 × 0.20 × 0.20 mm
*Z* = 2	

### Data collection

**Table:**

Bruker APEXII CCD area-detector diffractometer	4326 independent reflections
Radiation source: fine-focus sealed tube	3432 reflections with *I* > 2σ(*I*)
graphite	*R*_int_ = 0.020
φ and ω scans	θ_max_ = 26.0°, θ_min_ = 1.7°
Absorption correction: ψ scan (*SADABS*; Sheldrick, 1996)	*h* = −11→10
*T*_min_ = 0.758, *T*_max_ = 0.792	*k* = −11→12
12095 measured reflections	*l* = −30→28

### Refinement

**Table:**

Refinement on *F*^2^	Primary atom site location: structure-invariant direct methods
Least-squares matrix: full	Secondary atom site location: difference Fourier map
*R*[*F*^2^ > 2σ(*F*^2^)] = 0.031	Hydrogen site location: inferred from neighbouring sites
*wR*(*F*^2^) = 0.085	H-atom parameters constrained
*S* = 1.03	*w* = 1/[σ^2^(*F*_o_^2^) + (0.0468*P*)^2^ + 0.3207*P*] where *P* = (*F*_o_^2^ + 2*F*_c_^2^)/3
4326 reflections	(Δ/σ)_max_ = 0.001
275 parameters	Δρ_max_ = 0.21 e Å^−^^3^
0 restraints	Δρ_min_ = −0.28 e Å^−^^3^

### Special details

**Table:**

Geometry. All e.s.d.'s (except the e.s.d. in the dihedral angle between two l.s. planes) are estimated using the full covariance matrix. The cell e.s.d.'s are taken into account individually in the estimation of e.s.d.'s in distances, angles and torsion angles; correlations between e.s.d.'s in cell parameters are only used when they are defined by crystal symmetry. An approximate (isotropic) treatment of cell e.s.d.'s is used for estimating e.s.d.'s involving l.s. planes.
Refinement. Refinement of *F*^2^ against ALL reflections. The weighted *R*-factor *wR* and goodness of fit *S* are based on *F*^2^, conventional *R*-factors *R* are based on *F*, with *F* set to zero for negative *F*^2^. The threshold expression of *F*^2^ > σ(*F*^2^) is used only for calculating *R*-factors(gt) *etc*. and is not relevant to the choice of reflections for refinement. *R*-factors based on *F*^2^ are statistically about twice as large as those based on *F*, and *R*- factors based on ALL data will be even larger.

### Fractional atomic coordinates and isotropic or equivalent isotropic displacement parameters (Å^2^)

**Table:**

	*x*	*y*	*z*	*U*_iso_*/*U*_eq_	
Zn1	1.13033 (2)	0.90381 (2)	0.006145 (10)	0.05090 (10)	
O1	0.83151 (18)	0.93234 (15)	−0.05487 (7)	0.0678 (4)	
O2	1.01833 (16)	0.78450 (16)	−0.04910 (6)	0.0653 (4)	
O3	0.98300 (16)	0.84236 (16)	0.06369 (6)	0.0635 (4)	
O4	0.79517 (17)	0.98974 (15)	0.05820 (6)	0.0662 (4)	
N1	1.31222 (17)	0.79423 (16)	0.02637 (7)	0.0513 (4)	
N2	1.67263 (18)	0.53691 (18)	0.05771 (7)	0.0564 (4)	
C1	0.8638 (2)	0.8943 (2)	0.08022 (9)	0.0531 (5)	
C2	0.8981 (2)	0.8279 (2)	−0.06917 (9)	0.0535 (5)	
C3	0.8268 (2)	0.7505 (2)	−0.11421 (8)	0.0508 (5)	
C4	0.8823 (3)	0.6275 (2)	−0.13030 (9)	0.0609 (6)	
H4	0.9639	0.5903	−0.1121	0.073*	
C5	0.8182 (3)	0.5598 (3)	−0.17289 (10)	0.0756 (7)	
H5	0.8576	0.4774	−0.1832	0.091*	
C6	0.6971 (4)	0.6111 (3)	−0.20062 (12)	0.0881 (8)	
C7	0.6411 (3)	0.7325 (3)	−0.18418 (12)	0.0934 (9)	
H7	0.5589	0.7689	−0.2023	0.112*	
C8	0.7040 (3)	0.8013 (3)	−0.14147 (10)	0.0724 (6)	
H8	0.6632	0.8828	−0.1309	0.087*	
C9	0.8005 (2)	0.8393 (2)	0.13138 (8)	0.0540 (5)	
C10	0.6700 (3)	0.8879 (3)	0.15302 (11)	0.0788 (7)	
H10	0.6151	0.9527	0.1344	0.095*	
C11	0.6197 (3)	0.8414 (3)	0.20207 (12)	0.0911 (9)	
H11	0.5312	0.8757	0.2159	0.109*	
C12	0.6974 (3)	0.7456 (3)	0.23093 (10)	0.0764 (7)	
C13	0.8222 (3)	0.6931 (3)	0.20795 (10)	0.0796 (7)	
H13	0.8736	0.6245	0.2256	0.096*	
C14	0.8740 (3)	0.7392 (3)	0.15917 (9)	0.0699 (6)	
H14	0.9603	0.7019	0.1448	0.084*	
C15	1.4414 (2)	0.8023 (2)	−0.00023 (9)	0.0560 (5)	
H15	1.4486	0.8667	−0.0273	0.067*	
C16	1.5632 (2)	0.7230 (2)	0.00943 (9)	0.0558 (5)	
H16	1.6499	0.7354	−0.0104	0.067*	
C17	1.5585 (2)	0.62332 (19)	0.04898 (8)	0.0483 (5)	
C18	1.4256 (2)	0.6191 (2)	0.07871 (9)	0.0612 (6)	
H18	1.4165	0.5590	0.1072	0.073*	
C19	1.3102 (2)	0.7026 (2)	0.06594 (9)	0.0626 (6)	
H19	1.2234	0.6955	0.0862	0.075*	
C20	1.6662 (3)	0.4385 (3)	0.10068 (11)	0.0752 (7)	
H20A	1.6706	0.4833	0.1350	0.113*	
H20B	1.7495	0.3781	0.0978	0.113*	
H20C	1.5743	0.3889	0.0977	0.113*	
C21	1.8086 (2)	0.5430 (2)	0.02665 (10)	0.0648 (6)	
H21A	1.7837	0.5548	−0.0109	0.097*	
H21B	1.8637	0.4611	0.0313	0.097*	
H21C	1.8685	0.6172	0.0389	0.097*	
C22	0.6270 (6)	0.5356 (5)	−0.24752 (17)	0.166 (2)	
H22A	0.6512	0.5800	−0.2807	0.249*	
H22B	0.5203	0.5335	−0.2434	0.249*	
H22C	0.6651	0.4456	−0.2482	0.249*	
C23	0.6490 (4)	0.7010 (4)	0.28691 (11)	0.1126 (12)	
H23A	0.6595	0.6054	0.2900	0.169*	
H23B	0.5463	0.7254	0.2923	0.169*	
H23C	0.7108	0.7440	0.3138	0.169*	

### Atomic displacement parameters (Å^2^)

**Table:**

	*U*^11^	*U*^22^	*U*^33^	*U*^12^	*U*^13^	*U*^23^
Zn1	0.04359 (14)	0.04260 (15)	0.06657 (18)	0.00739 (9)	0.00287 (10)	−0.00171 (11)
O1	0.0702 (10)	0.0516 (9)	0.0817 (11)	0.0032 (7)	−0.0006 (8)	−0.0136 (8)
O2	0.0597 (9)	0.0620 (10)	0.0741 (10)	0.0013 (7)	−0.0071 (8)	−0.0082 (8)
O3	0.0556 (9)	0.0614 (9)	0.0738 (10)	0.0026 (7)	0.0160 (7)	0.0037 (8)
O4	0.0654 (9)	0.0570 (9)	0.0766 (10)	0.0070 (7)	0.0126 (8)	0.0074 (8)
N1	0.0447 (9)	0.0450 (9)	0.0641 (10)	0.0063 (7)	0.0043 (8)	0.0011 (8)
N2	0.0457 (9)	0.0522 (10)	0.0712 (11)	0.0056 (8)	−0.0056 (8)	0.0082 (9)
C1	0.0511 (11)	0.0467 (12)	0.0616 (13)	−0.0043 (9)	0.0032 (10)	−0.0070 (10)
C2	0.0528 (12)	0.0482 (12)	0.0598 (12)	−0.0067 (9)	0.0100 (10)	−0.0005 (10)
C3	0.0499 (11)	0.0485 (11)	0.0540 (11)	−0.0045 (9)	0.0080 (9)	0.0017 (9)
C4	0.0607 (13)	0.0546 (13)	0.0676 (14)	0.0030 (10)	0.0008 (11)	−0.0032 (11)
C5	0.0865 (18)	0.0614 (15)	0.0790 (17)	0.0038 (13)	0.0002 (14)	−0.0168 (13)
C6	0.098 (2)	0.085 (2)	0.0801 (18)	−0.0004 (16)	−0.0184 (16)	−0.0201 (15)
C7	0.092 (2)	0.094 (2)	0.094 (2)	0.0121 (16)	−0.0355 (16)	−0.0111 (17)
C8	0.0743 (15)	0.0655 (15)	0.0772 (16)	0.0103 (12)	−0.0059 (13)	−0.0044 (13)
C9	0.0538 (11)	0.0495 (12)	0.0587 (12)	−0.0059 (9)	0.0051 (10)	−0.0104 (10)
C10	0.0797 (17)	0.0779 (18)	0.0793 (17)	0.0133 (13)	0.0220 (14)	0.0023 (14)
C11	0.0867 (19)	0.099 (2)	0.088 (2)	0.0087 (17)	0.0367 (16)	−0.0091 (18)
C12	0.0894 (19)	0.0836 (18)	0.0564 (14)	−0.0188 (15)	0.0126 (13)	−0.0142 (14)
C13	0.0849 (18)	0.0905 (19)	0.0635 (15)	−0.0005 (15)	0.0011 (13)	0.0089 (14)
C14	0.0683 (14)	0.0770 (16)	0.0646 (14)	0.0052 (12)	0.0088 (11)	0.0022 (13)
C15	0.0511 (11)	0.0451 (11)	0.0720 (14)	0.0026 (9)	0.0079 (10)	0.0118 (10)
C16	0.0430 (10)	0.0507 (12)	0.0738 (14)	0.0031 (9)	0.0099 (9)	0.0090 (11)
C17	0.0431 (10)	0.0436 (11)	0.0582 (12)	−0.0005 (8)	−0.0043 (9)	−0.0020 (9)
C18	0.0523 (12)	0.0662 (14)	0.0651 (14)	0.0066 (10)	0.0034 (10)	0.0185 (11)
C19	0.0475 (11)	0.0728 (15)	0.0679 (14)	0.0083 (10)	0.0120 (10)	0.0096 (12)
C20	0.0672 (15)	0.0716 (16)	0.0866 (17)	0.0099 (12)	−0.0103 (13)	0.0199 (14)
C21	0.0490 (12)	0.0591 (13)	0.0863 (16)	0.0112 (10)	0.0010 (11)	−0.0007 (12)
C22	0.186 (5)	0.159 (4)	0.150 (4)	0.019 (3)	−0.084 (3)	−0.075 (3)
C23	0.145 (3)	0.133 (3)	0.0599 (16)	−0.024 (2)	0.0253 (18)	−0.0083 (18)

### Geometric parameters (Å, °)

**Table:**

Zn1—Zn1^i^	3.0287 (6)	C12—C13	1.362 (4)
Zn1—O1^i^	2.0564 (16)	C12—C23	1.523 (4)
Zn1—O4^i^	2.0320 (16)	C13—C14	1.377 (3)
O1—Zn1^i^	2.0564 (16)	C13—H13	0.9300
O2—Zn1	2.0626 (15)	C14—H14	0.9300
O3—Zn1	2.0438 (15)	C15—N1	1.337 (3)
O4—Zn1^i^	2.0320 (16)	C15—C16	1.365 (3)
N1—Zn1	2.0160 (16)	C15—H15	0.9300
C1—O4	1.253 (2)	C16—C17	1.396 (3)
C1—O3	1.256 (2)	C16—H16	0.9300
C1—C9	1.497 (3)	C17—N2	1.350 (2)
C2—O1	1.252 (3)	C17—C18	1.404 (3)
C2—O2	1.255 (3)	C18—C19	1.359 (3)
C2—C3	1.492 (3)	C18—H18	0.9300
C3—C8	1.378 (3)	C19—N1	1.340 (3)
C3—C4	1.383 (3)	C19—H19	0.9300
C4—C5	1.372 (3)	C20—N2	1.449 (3)
C4—H4	0.9300	C20—H20A	0.9600
C5—C6	1.373 (4)	C20—H20B	0.9600
C5—H5	0.9300	C20—H20C	0.9600
C6—C7	1.373 (4)	C21—N2	1.446 (3)
C6—C22	1.513 (4)	C21—H21A	0.9600
C7—C8	1.375 (4)	C21—H21B	0.9600
C7—H7	0.9300	C21—H21C	0.9600
C8—H8	0.9300	C22—H22A	0.9600
C9—C14	1.375 (3)	C22—H22B	0.9600
C9—C10	1.376 (3)	C22—H22C	0.9600
C10—C11	1.379 (4)	C23—H23A	0.9600
C10—H10	0.9300	C23—H23B	0.9600
C11—C12	1.376 (4)	C23—H23C	0.9600
C11—H11	0.9300		
			
O1^i^—Zn1—Zn1^i^	71.24 (5)	C9—C10—H10	119.7
O1^i^—Zn1—O2	157.33 (6)	C11—C10—H10	119.7
O2—Zn1—Zn1^i^	86.09 (5)	C12—C11—C10	121.5 (3)
O3—Zn1—Zn1^i^	76.09 (5)	C12—C11—H11	119.2
O3—Zn1—O1^i^	86.16 (7)	C10—C11—H11	119.2
O3—Zn1—O2	88.75 (6)	C13—C12—C11	117.4 (2)
O4^i^—Zn1—Zn1^i^	81.30 (5)	C13—C12—C23	120.6 (3)
O4^i^—Zn1—O1^i^	89.51 (7)	C11—C12—C23	121.9 (3)
O4^i^—Zn1—O2	86.67 (7)	C12—C13—C14	121.5 (3)
O4^i^—Zn1—O3	157.18 (6)	C12—C13—H13	119.2
N1—Zn1—Zn1^i^	169.82 (5)	C14—C13—H13	119.2
N1—Zn1—O1^i^	99.01 (7)	C9—C14—C13	121.2 (2)
N1—Zn1—O2	103.64 (7)	C9—C14—H14	119.4
N1—Zn1—O3	100.83 (7)	C13—C14—H14	119.4
N1—Zn1—O4^i^	101.98 (7)	N1—C15—C16	124.71 (19)
C2—O1—Zn1^i^	138.60 (15)	N1—C15—H15	117.6
C2—O2—Zn1	118.06 (14)	C16—C15—H15	117.6
C1—O3—Zn1	131.15 (15)	C15—C16—C17	120.32 (19)
C1—O4—Zn1^i^	125.07 (14)	C15—C16—H16	119.8
C15—N1—C19	114.85 (17)	C17—C16—H16	119.8
C15—N1—Zn1	122.82 (14)	N2—C17—C16	122.49 (18)
C19—N1—Zn1	122.25 (13)	N2—C17—C18	122.56 (19)
C17—N2—C21	121.69 (18)	C16—C17—C18	114.95 (18)
C17—N2—C20	120.89 (18)	C19—C18—C17	120.2 (2)
C21—N2—C20	117.35 (18)	C19—C18—H18	119.9
O4—C1—O3	125.7 (2)	C17—C18—H18	119.9
O4—C1—C9	117.31 (19)	N1—C19—C18	124.83 (19)
O3—C1—C9	117.00 (19)	N1—C19—H19	117.6
O1—C2—O2	125.5 (2)	C18—C19—H19	117.6
O1—C2—C3	116.2 (2)	N2—C20—H20A	109.5
O2—C2—C3	118.28 (19)	N2—C20—H20B	109.5
C8—C3—C4	118.0 (2)	H20A—C20—H20B	109.5
C8—C3—C2	120.5 (2)	N2—C20—H20C	109.5
C4—C3—C2	121.5 (2)	H20A—C20—H20C	109.5
C5—C4—C3	120.7 (2)	H20B—C20—H20C	109.5
C5—C4—H4	119.7	N2—C21—H21A	109.5
C3—C4—H4	119.7	N2—C21—H21B	109.5
C4—C5—C6	121.4 (2)	H21A—C21—H21B	109.5
C4—C5—H5	119.3	N2—C21—H21C	109.5
C6—C5—H5	119.3	H21A—C21—H21C	109.5
C7—C6—C5	117.9 (3)	H21B—C21—H21C	109.5
C7—C6—C22	121.1 (3)	C6—C22—H22A	109.5
C5—C6—C22	121.0 (3)	C6—C22—H22B	109.5
C6—C7—C8	121.4 (3)	H22A—C22—H22B	109.5
C6—C7—H7	119.3	C6—C22—H22C	109.5
C8—C7—H7	119.3	H22A—C22—H22C	109.5
C7—C8—C3	120.7 (2)	H22B—C22—H22C	109.5
C7—C8—H8	119.7	C12—C23—H23A	109.5
C3—C8—H8	119.7	C12—C23—H23B	109.5
C14—C9—C10	117.6 (2)	H23A—C23—H23B	109.5
C14—C9—C1	120.44 (19)	C12—C23—H23C	109.5
C10—C9—C1	122.0 (2)	H23A—C23—H23C	109.5
C9—C10—C11	120.7 (3)	H23B—C23—H23C	109.5
			
O1—C2—C3—C8	6.7 (3)	C16—C15—N1—Zn1	−174.77 (17)
O2—C2—C3—C8	−172.9 (2)	C18—C19—N1—C15	−1.8 (3)
O1—C2—C3—C4	−174.11 (19)	C18—C19—N1—Zn1	174.94 (19)
O2—C2—C3—C4	6.3 (3)	C16—C17—N2—C21	0.5 (3)
C8—C3—C4—C5	1.3 (3)	C18—C17—N2—C21	−180.0 (2)
C2—C3—C4—C5	−177.9 (2)	C16—C17—N2—C20	177.3 (2)
C3—C4—C5—C6	−0.4 (4)	C18—C17—N2—C20	−3.2 (3)
C4—C5—C6—C7	−0.4 (5)	O2—C2—O1—Zn1^i^	9.7 (4)
C4—C5—C6—C22	−179.9 (3)	C3—C2—O1—Zn1^i^	−169.87 (15)
C5—C6—C7—C8	0.2 (5)	O1—C2—O2—Zn1	−7.3 (3)
C22—C6—C7—C8	179.8 (4)	C3—C2—O2—Zn1	172.26 (13)
C6—C7—C8—C3	0.7 (5)	O4—C1—O3—Zn1	11.8 (3)
C4—C3—C8—C7	−1.5 (4)	C9—C1—O3—Zn1	−166.70 (13)
C2—C3—C8—C7	177.8 (2)	O3—C1—O4—Zn1^i^	−5.9 (3)
O4—C1—C9—C14	−176.7 (2)	C9—C1—O4—Zn1^i^	172.51 (13)
O3—C1—C9—C14	1.9 (3)	C15—N1—Zn1—O4^i^	0.20 (17)
O4—C1—C9—C10	1.8 (3)	C19—N1—Zn1—O4^i^	−176.33 (16)
O3—C1—C9—C10	−179.6 (2)	C15—N1—Zn1—O3	−179.00 (16)
C14—C9—C10—C11	2.9 (4)	C19—N1—Zn1—O3	4.47 (18)
C1—C9—C10—C11	−175.6 (2)	C15—N1—Zn1—O1^i^	−91.22 (17)
C9—C10—C11—C12	0.0 (5)	C19—N1—Zn1—O1^i^	92.25 (17)
C10—C11—C12—C13	−3.3 (5)	C15—N1—Zn1—O2	89.65 (17)
C10—C11—C12—C23	175.5 (3)	C19—N1—Zn1—O2	−86.88 (17)
C11—C12—C13—C14	3.7 (4)	C15—N1—Zn1—Zn1^i^	−107.6 (3)
C23—C12—C13—C14	−175.1 (3)	C19—N1—Zn1—Zn1^i^	75.8 (3)
C10—C9—C14—C13	−2.6 (4)	C1—O3—Zn1—N1	161.67 (19)
C1—C9—C14—C13	176.0 (2)	C1—O3—Zn1—O4^i^	−16.3 (3)
C12—C13—C14—C9	−0.8 (4)	C1—O3—Zn1—O1^i^	63.22 (19)
N1—C15—C16—C17	0.9 (3)	C1—O3—Zn1—O2	−94.7 (2)
C15—C16—C17—N2	175.8 (2)	C1—O3—Zn1—Zn1^i^	−8.39 (18)
C15—C16—C17—C18	−3.8 (3)	C2—O2—Zn1—N1	−179.95 (14)
N2—C17—C18—C19	−175.6 (2)	C2—O2—Zn1—O4^i^	−78.43 (16)
C16—C17—C18—C19	3.9 (3)	C2—O2—Zn1—O3	79.21 (15)
C17—C18—C19—N1	−1.2 (4)	C2—O2—Zn1—O1^i^	2.3 (3)
C16—C15—N1—C19	2.0 (3)	C2—O2—Zn1—Zn1^i^	3.07 (15)

Symmetry codes: (i) −*x*+2, −*y*+2, −*z*.

### Hydrogen-bond geometry (Å, °)

**Table:**

*D*—H···*A*	*D*—H	H···*A*	*D*···*A*	*D*—H···*A*
C21—H21C···O3^ii^	0.96	2.54	3.484 (3)	168
C23—H23B···Cg1^iii^	0.96	2.99	3.925 (4)	165

Symmetry codes: (ii) *x*+1, *y*, *z*; (iii) *x*−1/2, −*y*+3/2, *z*+1/2.
